# Development of a simple method to measure static body weight distribution in neurologically and orthopedically normal mature small breed dogs

**DOI:** 10.1186/s12917-021-02808-x

**Published:** 2021-03-06

**Authors:** Jessica E. Linder, Stephanie Thomovsky, Jessica Bowditch, Mallory Lind, Kristine A. Kazmierczak, Gert J. Breur, Melissa J. Lewis

**Affiliations:** 1 Department of Clinical Sciences, College of Veterinary Medicine, Purdue University, 625 Harrison Street, IN 47907 West Lafayette, USA; 2 College of Veterinary Medicine, University of Florida, 2015 SW 16th Avenue, FL 32608 Gainesville, USA

**Keywords:** Body weight distribution, Canine, Chondrodystrophic, Digital scales, Spinal cord injury

## Abstract

**Background:**

Objective outcome measures capable of tracking different aspects of functional recovery in dogs with acute intervertebral disc herniation are needed to optimize physical rehabilitation protocols. Normal, pre-injury distribution of body weight in this population is unknown. The aims of this study were to quantify static weight distribution (SWD) using digital scales and to establish the feasibility of different scale methods in neurologically normal, mature, chondrodystrophic small breed dogs predisposed to intervertebral disc herniation.

**Results:**

Twenty-five healthy, mature dogs were enrolled with a mean age of 4.6 years (SD 2.7) and a mean total body weight of 11.5 kg (SD 3.6). SWD for the thoracic and pelvic limbs and between individual limbs was acquired in triplicate and expressed as a percentage of total body weight using commercially available digital scales in four combinations: two bathroom, two kitchen (with thoracic and pelvic limbs combined), four bathroom and four kitchen (with limbs measured individually). SWD was also obtained using a pressure sensing walkway for comparison to scale data. Feasibility for each method was determined and coefficients of variation were used to calculate inter-trial variability. Mean SWD values were compared between methods using an ANOVA. The two bathroom scales method had the highest feasibility and lowest inter-trial variability and resulted in mean thoracic and pelvic limb SWD of 63 % (SD 3 %) and 37 % (SD 3 %), respectively. Thoracic limb mean SWD was higher for the PSW compared to any of the scale methods (*p *< 0.0001).

**Conclusions:**

SWD in a population of healthy chondrodystrophic dogs was simple to obtain using inexpensive and readily available digital scales. This study generated SWD data for subsequent comparison to dogs recovering from acute intervertebral disc herniation.

## Background

Physical rehabilitation is increasingly being utilized in veterinary medicine with the goals of improving mobility, promoting return of normal functional status and enhancing overall quality of life [1, 2]. In dogs with acute SCI secondary to thoracolumbar IVDH, rehabilitation protocols are commonly recommended and incorporated as part of a multimodal approach to recovery [3]. Despite the widespread use of rehabilitation in this population, currently there are no standardized nor validated protocols regarding initiation, duration, type of modalities or exercises performed. Additionally, studies evaluating its benefits in dogs following SCI have reported mixed results [4–10].

A major challenge in defining the role of rehabilitation in acute SCI is the lack of validated outcome measures capable of objectively tracking changes throughout recovery. Multiple gait analysis tools have been developed in dogs, ranging from ordinal, open field scales to force platforms and PSW [11–20]. These techniques allow objective assessment of dynamic weight distribution and alterations from normal gait patterns, but can be difficult to apply in non-ambulatory dogs and variably require specialized equipment or training to perform successfully [11, 15–20]. Recently, a functional testing battery was validated to evaluate motor function in dogs recovering from SCI [21]. While these methods provide ways to broadly evaluate motor and ambulation, additional tools are needed to complement these methods and provide simple, reliable means to quantify other aspects of functional recovery in dogs undergoing rehabilitation.

In dogs with SCI secondary to thoracolumbar IVDH, forward shifting of center of pressure or BW and a widened thoracic limb base of support have been demonstrated while walking with or without assistance [15, 20]. These studies suggest increased loading of the thoracic limbs might occur as compensation for pelvic limb weakness. Measuring SWD, therefore, might provide an objective target to monitor during the recovery process. However, the standing distribution of BW between thoracic and pelvic limbs and among individual limbs is unknown in neurologically normal dogs predisposed to IVDH. SWD using commercially available digital scales has been reported in normal large breed dogs [22, 23] with 64 % of weight borne by the thoracic limbs [23]. Left to right pelvic limb asymmetric weight distribution has also been reported using digital scales in large breed dogs with osteoarthritis, compared to healthy controls [22]. Using digital bathroom scales to capture SWD has been suggested to be a simple outcome measure for use in dogs with osteoarthritis undergoing rehabilitation [22]. The feasibility of using digital scales to acquire SWD has not been established in normal smaller breed dogs.

The aims of this study were: to quantify SWD in neurologically normal chondrodystophic small breed dogs predisposed to Hansen type I IVDH, to establish the simplest and most reliable method of capturing weight distribution data in this population using four combinations of commercially available digital scales, and to compare values obtained with digital scales to those obtained using the PSW. We hypothesized that all digital scale methods would be feasible in small breed dogs and that they would be comparable to values obtained using the PSW.

## Results

### Study population

Twenty-five mature, small breed dogs were prospectively enrolled. Six Dachshunds, four Beagles, four Corgis, one Bassett Hound, one French Bulldog, one Shih Tzu, one Pug, and seven chondrodystrophic mixed breeds were included. The mean age of participants was 4.6 years (SD 2.7) and mean BW was 11.5 kg (SD 3.6). Thirteen of the dogs were males, and 12 were females. All dogs had normal physical, neurologic and orthopedic examinations.

### Feasibility and inter‐trial variability

All dogs participated willingly in testing and, after a brief period of acclimation and practice, were able to start data acquisition. The feasibility of each acquisition method and the BW ranges of dogs that successfully participated in each method are listed in Table 1. The B2 and PSW methods has the highest feasibility scores, both of which could be performed in 24/25 dogs (96 %). Two dogs did not complete all test procedures; in one dog, PSW data was not obtained due to non-cooperative behavior and in another, technical difficulty with the B2 method (transient scale malfunction) prevented acquiring data. Using the B4 method was not possible in smaller dogs (< 8.4 kg) due to values not registering for individual limbs (below the 1.4kg limit of detection). The K2 and K4 methods were not possible in larger dogs (> 8.6kg, > 13.1kg, respectively) due to exceeding the upper weight range reported for these scales (5kg).

**Table 1:** Feasibility and BW ranges for each measurement method

Measurement Method	Feasibility	BW Range (kg)
PSW	24/25 (96 %)	5.3-19.05
B2	24/25 (96 %)	5.3-19.05
B4	13/25 (52 %)	8.4-19.05
K2	5/25 (20 %)	5.3–8.6
K4	15/25 (60 %)	5.3–13.1

Variability across trials for each scale method is presented in Table 2. The B2 and K2 methods were more reliable compared to B4 and K4 methods. Using the two scale methods, coefficients of variation for thoracic and pelvic limb measurements between trials were less than 10 %. With the four scale methods, inter-trial variability in measurements for each individual limb was greater and ranged from 9 to 21 %.

**Table 2:** Coefficients of variation for each scale method

Measurement Method	Thoracic Limbs		Pelvic Limbs	
B2	4.8 % (1.7 %-12.7 %)		7.6 % (1.7 %-18.2 %)	
K2	3.8 % (1.2 %-1.3 %)		4.5 % (2.3 %-7.2 %)	
	Left Thoracic Limb	Right Thoracic Limb	Left Pelvic Limb	Right Pelvic Limb
B4	17.1 % (4.8 %-38.1 %)	14.6 % (0 %-29.5 %)	21.1 % (5.8 %-48.3 %)	14.9 % (0 %-37.0 %)
K4	17.3 % (2.7 %-31.2 %)	14.4 % (2.5 %-29.1 %)	9.26 % (0 %-24.7 %)	9.9 % (0 %-19.9 %)

### Static weight distribution

**Fig. 1:**
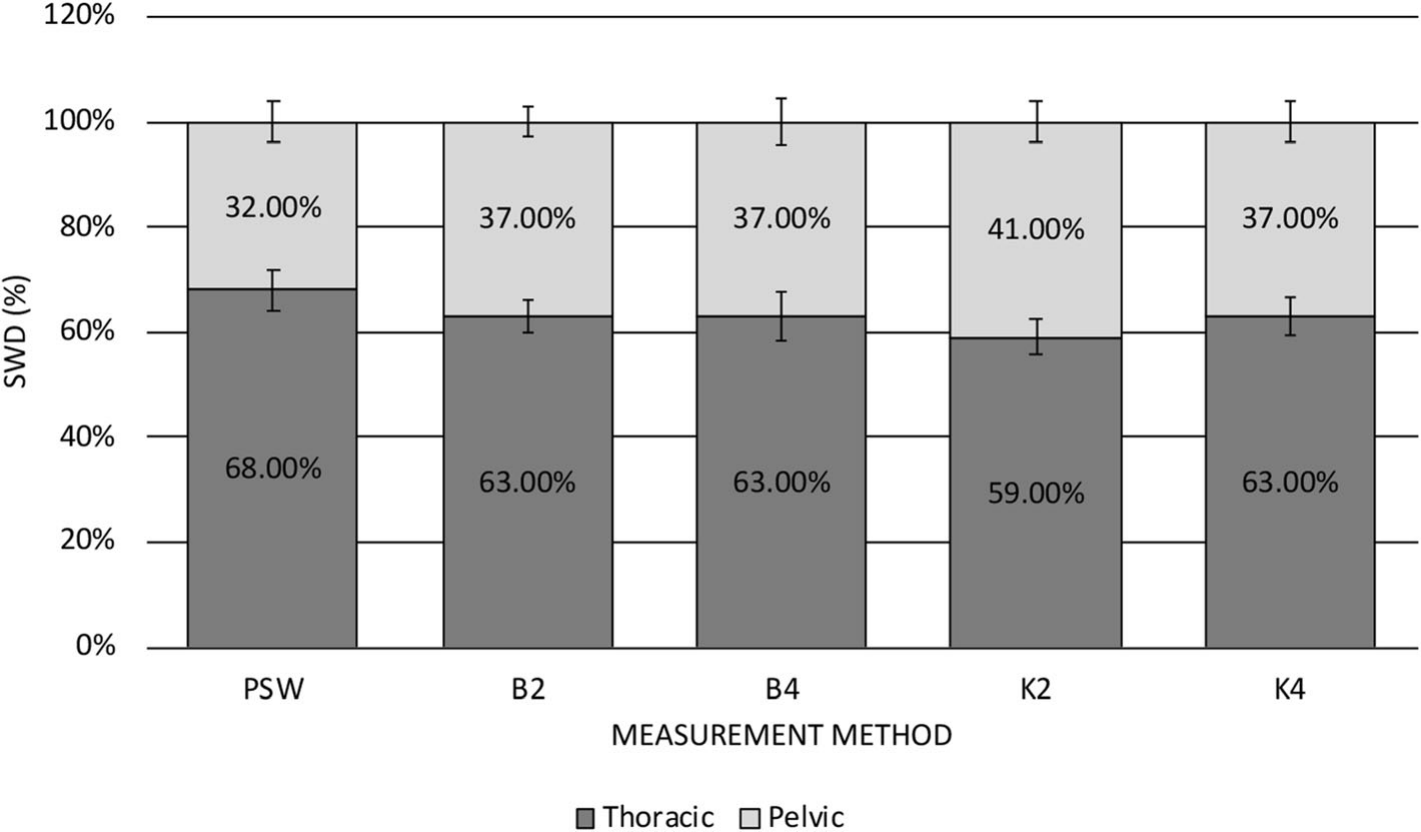
Mean thoracic to pelvic limb SWD as a percentage of total BW for each measurement method

**Fig. 2:**
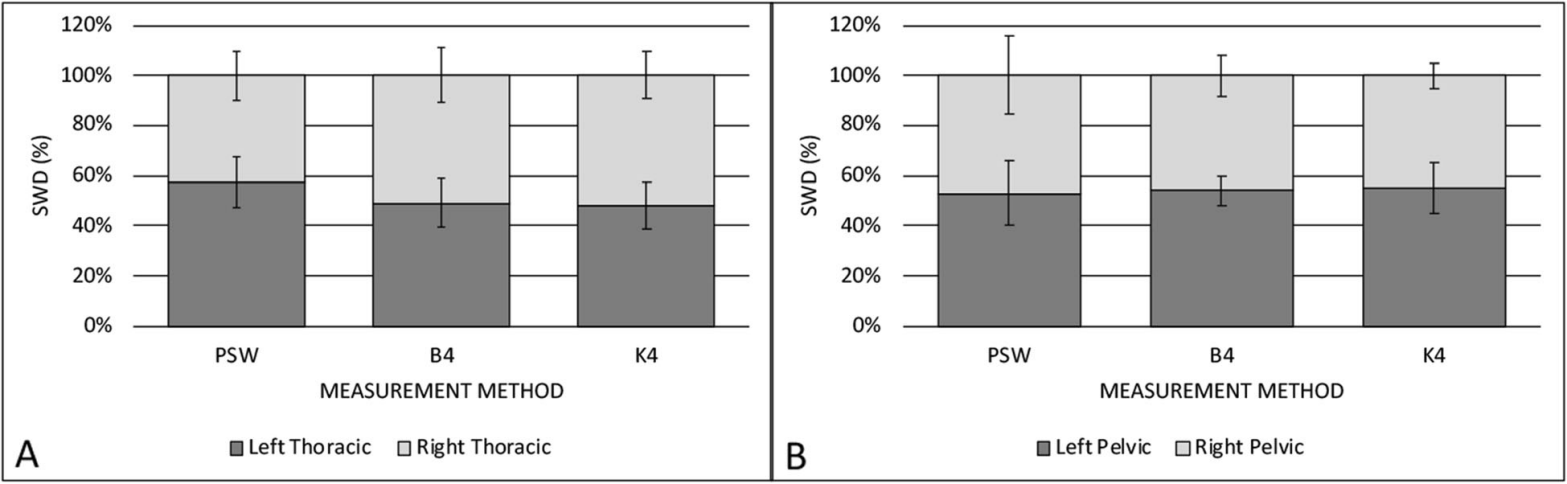
Mean left to right SWD for **a**) thoracic and **b**) pelvic limbs as a percentage of total thoracic or pelvic limb BW, respectively, for each measurement method (PSW, B4, K4)

Mean thoracic to pelvic limb SWD for each method is depicted in Fig. 1. Across the scale methods, the mean SWD for the thoracic limbs ranged from 59 to 63 % (SD 3.0–4.0 %) and the pelvic limbs ranged from 37 to 41 % (SD 3.0-4.5 %). Mean thoracic and pelvic limb SWD for the PSW were 68 % (SD 4.0 %) and 32 % (SD 4.0 %), respectively. The thoracic limb SWD was significantly higher for values obtained on the PSW compared to any of the scale methods (*p* < 0.0001). For the methods in which individual limb values were obtained (B4, K4, PSW), mean left to right SWD between the thoracic limbs and between pelvic limbs are outlined in Fig. 2. The mean left to right asymmetry between the thoracic limbs was 8.7 % (SD 7.5 %), 8.6 % (SD 6.3 %) and 12.8 % (SD 9.1 %) for the B4, K4 and PSW measurement methods, respectively. The mean left to right asymmetry for the pelvic limbs between the B4, K4 and PSW methods was 3.7 % (SD 2.9 %), 4.3 % (SD 3.6 %) and 6.0 % (SD 5.2 %), respectively. Left to right asymmetry values for either the thoracic or pelvic limbs were not significantly different between methods (*p* > 0.1).

## Discussion

Our results demonstrate that SWD was feasible to obtain using commercially available digital scales in a population of neurologically normal dogs ranging from 5 to 20 kilograms in BW. Using two digital bathroom scales (B2) was the simplest and most reliable technique, and resulted in a thoracic limb to pelvic limb SWD of 63–37 %. The other scale methods might be useful in specific scenarios such as using the kitchen scales for very small dogs, or using one of the four scale methods where capturing left to right asymmetry is important.

We tested four different digital scale combinations and the B2 method was by far the most feasible method across a broad BW range of 5 to 20kg. While the B4 and K4 methods also provided adequate feasibility, they were limited to dogs of higher and lower BW, respectively. This reflects the lower and upper weight ranges of these commercially available scales, reported to be a 1.4kg minimum for the bathroom scales and a 5kg maximum for the kitchen scales. The K2 method was even more limited to smaller dogs weighing less than approximately 9kg. A high level of feasibility of bathroom scales to measure SWD has been previously reported in normal dogs and dogs with osteoarthritis [22]. While the prior study evaluated large breed dogs weighing greater than 20kg, our results confirmed that these techniques are straightforward to employ across dogs of different sizes.

We recruited dogs that were amenable to handling and, therefore, encountered only a single dog in which measurements were limited by a behavioral issue (measurements were initially obtained easily but the dog tired of the handling and became aggressive). To facilitate cooperation and tolerance, we allowed all dogs several minutes to acclimate to the procedures and to having their limbs manipulated before starting to collect measurements and took breaks as needed. It is possible that behavioral issues will be more apparent in dogs that might be painful secondary to IVDH or surgery. However, similar measurements were obtained in 41/43 dogs with osteoarthritis with only two dogs unable to participate due to behavioral issues [22]. This suggests that feasibility when translated to a clinical population would still be expected to be high especially since all scale methods and the PSW would not typically need to be performed in clinical patients.

Variability between trials made in triplicate was lowest for the two scale methods, B2 or K2. While there was some inter-individual variation (coefficients of variation ranging from 1.7 to 18.2 %), the overall variability between trials was less than 10 % for both methods. It was easiest to position the dogs appropriately when the thoracic and pelvic limbs were each contained on a single scale and the dogs appeared most comfortable, resulting in minimal cranial to caudal weight shifting while performing the task. Our results are similar to Levine et al. where SWD was evaluated in 10 healthy, large breed dogs [23]. The reported mean coefficient of variation across all measurements (also made in triplicate) was 4.0 % (0–24 %); coefficients of variation for the thoracic and pelvic limbs were reported to be 14 % and 13 %, respectively [23].

With the B4 or K4 methods, dogs appeared to stand squarely; however, any slight shift in position such as mild head movements, likely contributed to greater variation between trials. This variability ranged from approximately 10–21 % across dogs and 0–48 % between trials in individual dogs. Similarly, left and right pelvic limb SWD measurements have been previously reported in large breed dogs to have an overall reliability of 76 % including 61 % for normal dogs and 79 % for the osteoarthritis group [22]. The authors suggested the lower test-retest repeatability in control dogs might be explained by a lack of need to focus on weight bearing between limbs in a normal dog. While we made every attempt to ensure dogs were standing still and squarely, no dogs were specifically trained to stand. Small, random shifting between limbs might be normal in healthy dogs. Considering feasibility and variability together, the B2 method provided the simplest and most robust means to measure SWD in this population of normal, small breed dogs. The other methods were adequate and offer specific situations in which they might be a useful adjunct to the B2 method.

Across all scale methods, there was a mean thoracic limb SWD of 59–63 % and mean pelvic limb SWD of 37–41 % of total BW. This compares favorably to the distribution reported in normal, large breed dogs, 64–36 %, respectively [22, 23]. Interestingly, using the PSW, an established means of measuring dynamic weight distribution in dogs, we found a mean thoracic to pelvic limb SWD of 68–32 %. This was significantly different from our scale methods with greater weight borne on the thoracic limbs. The reason for this difference is not clear, but it might relate to the manner of testing. For the PSW, dogs were commonly walked and stopped partway across the mat for testing. Limbs were still adjusted as needed to ensure dogs were square, but walking first might have led to artificially increased thoracic limb weight bearing upon stopping, or dogs might have leaned forward slightly in anticipation of starting to walk forward again. Alternatively, since the walkway is a continuous surface, it is possible their stance on the PSW was the most natural and therefore more accurate.

For the four scale methods (B4 or K4), the mean left to right asymmetry in SWD between thoracic limbs was approximately 8 % +/-7 % and for the pelvic limbs was approximately 4 % +/-3 %. This is compatible with prior studies in normal large breed dogs showing mild asymmetry between pelvic limbs when standing (3.3 % +/- 2.7 %) and in limb biomechanics when trotting [22, 24]. It is possible the left to right difference in our population reflected underlying disease; however, all of the dogs had normal orthopedic and neurologic examinations and no history of prior orthopedic or neurologic abnormalities. The greater degree of asymmetry observed in the thoracic limbs might be directly related to increased distribution of weight on these limbs or be attributed to head and neck position. Other contributing factors might include right or left dominance, similar to handedness in people, or small conformational discrepancies between limbs, which have been reported in dogs [24–26]. In general, our data support that mildly asymmetrical SWD occurs in normal dogs and should be taken into consideration when interpreting individual limb values in a clinically abnormal population.

The SWD data obtained in this population of normal dogs will allow for subsequent comparison to dogs recovering from IVDH, the most common cause of acute thoracolumbar SCI in dogs [27]. While it has been reported that more than half of neurosurgeons now recommend post-operative rehabilitation for dogs with acute thoracolumbar IVDH, additional validated outcome measures are needed to evaluate the role of rehabilitation in dogs recovering from SCI [3]. There are a number of evaluation methods currently available to monitor changes in gait [11–20]. Several of these gait scales have been shown to be reliable across raters with broad experience levels [28]. A battery of neurologic function tests that broadly assesses motor function called the FINFUN was also recently developed and validated in a group of dogs recovering from SCI [21]. However, there is a paucity of objective measures to quantify and track other aspects of functional recovery, such as weight distribution.

Abnormal weight shifting has been suggested in dogs with thoracolumbar SCI [15, 18, 20]. Increased forward loading of weight puts excess strain on cervical and thoracic limb muscles and joints and might cause myofascial pain, exacerbate osteoarthritis or otherwise negatively impact mobility. Decreased loading of abnormal limbs has been suggested to contribute to widespread nervous system and musculoskeletal changes including abnormal coordination, altered proprioception, impaired peripheral nerve health, muscle atrophy and weakness, decreased bone density and decreased overall joint, ligament and tendon health [29–33]. We anticipate that quantifying SWD will complement gait analysis tools and provide an additional target that might be useful in the periodic monitoring of neurologically abnormal patients undergoing rehabilitation.

## Conclusions

We developed a simple, objective method to quantify SWD in neurologically normal, mature, chrondrodystrophic small breed dogs. Using readily available and inexpensive digital scales, we demonstrated that measurement of thoracic to pelvic limb SWD is feasible, practical and can be easily implemented in any clinical setting. These results provide the foundation to compare to neurologically abnormal dogs recovering from acute thoracolumbar IVDH and to continue to develop this technique as an objective outcome measure for use in dogs rehabilitating from SCI.

## Methods

### Study population

Dogs were recruited through the Purdue University College of Veterinary Medicine listserv, the Purdue University Center for Comparative Translational Research Veterinary Clinical Trials website and advertisement in the reception area of the Veterinary Teaching Hospital. In order to participate, dogs had to be 1–10 years of age, weigh < 20 kg, and be systemically healthy with no history of neurologic or orthopedic abnormalities. For future data comparison to dogs with SCI, we targeted chondrodystrophic breeds or breed mixes predisposed to Hansen type I IVDH. General physical, neurologic and orthopedic exams were performed in all dogs (JEL, MJL). Dogs were excluded if there was evidence of neurologic or orthopedic disease. Informed consent was obtained from all owners and procedures were approved by and conducted in accordance with the Purdue University Animal Care and Use Committee (Protocol #1804001742).

### Digital scales

**Fig. 3:**
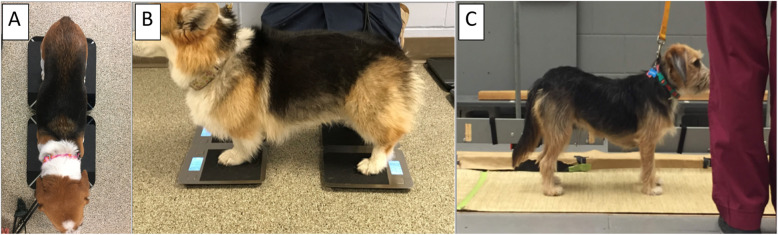
**a**-**c** Depictions of proper stance for **a**) the two scale (B2 and K2), **b**) four scale (B4 and K4) and **c**) PSW methods

Dogs were weighed in a standing position on factory calibrated, commercially available digital bathroom scales^1^ (range 1.4 to 200 kg, 0.1 kg accuracy) and digital kitchen scales^2^ (range 1 g to 5 kg, 1 g accuracy). A non-slip surface^3^ was applied to the top of each scale to facilitate ease of standing but they were otherwise unmodified. Dogs were acclimated to the procedure for several minutes before officially recording data. Measurements were obtained using four combinations of the scales: B2, B4, K2, and K4. The order of acquisition for the scale methods was randomly chosen between dogs. For the two scale methods (B2 or K2), the thoracic limbs were placed centrally on one scale, and pelvic limbs were placed centrally on a second scale (Fig. 3a). For the four scale methods (B4 or K4), one limb was placed in the center of each scale (Fig. 3b).

During data collection, dogs were required to stand squarely looking forward and to remain still during testing without manual correction or support. Brief rest breaks between measurement methods were given as needed. Three trials were performed for each scale combination in each dog. At least two people performed each trial observing and recording the values (in kilograms) for each limb or combination of limbs simultaneously.

Feasibility scores were generated for each stance trial for each combination of scales. A feasibility score of 1 was defined as standing appropriately for at least three seconds during data acquisition. A feasibility score of 0 was designated for dogs unable or unwilling to stand squarely for three seconds or when the scale was unable to register a numeric value. If a feasibility score of 1 was obtained for three trials, the method was considered feasible in that dog. If a feasibility score of 1 was not obtained in three separate trials, data from that scale combination was excluded from further analysis for that dog. Overall feasibility for each scale method was reported as the proportion of individual dogs in which the method was feasible relative to the total number of participants.

For each feasible method in a given dog, SWD was calculated as the mean weight distributed to each limb or pair of limbs across the three trials and reported as a percentage of total BW. For the four scale methods (B4 or K4), the SWD for each limb was obtained; SWD for the thoracic and pelvic limb pairs was determined by combining values for left and right limbs of the pair. To evaluate symmetry between pairs of limbs, left and right limb SWD values were also reported as a percentage of the thoracic limb or pelvic limb BW, respectively. For the two scale methods (B2 or K2), SWD was only calculated for thoracic limbs and pelvic limbs combined.

### Pressure sensing walkway

Using the Tekscan^4^^,^ pressure measurement system PSW, SWD was obtained in each dog for comparison to the four digital scale methods. The order relative to digital scale measurements was randomly chosen. Dogs were either placed in a standing position or walked along the gait analysis runway and stopped when they were on the pressure sensitive portion of the walkway. As with the digital scales, dogs were required to stand still for at least three seconds, positioned squarely and looking forward with all four paws contained within the PSW. Using these criteria and the standard data acquisition protocol established for the Purdue Animal Gait Laboratory, 10 to 12 trials were performed for each dog. Video footage and digitized maps of the dog’s feet were reviewed for each trial and rated as valid or not by at least two observers (JEL, MJL, KAK) (Fig. 3c). Trials were considered valid if the dog was standing as outlined above and all four paws registered on the digitized map. The PSW method received a feasibility score of 1 in which at least six valid trials were obtained. If a feasibility score of 1 was not achieved, then PSW data was not evaluated for that dog. Overall feasibility for the PSW was reported as the proportion of dogs in which the method was feasible relative to the total number of participants.

Six valid trials were analyzed for each dog using Tekscan Animal Walkway Software and formulas generated in a commercially available spreadsheet program. Vertical force data (in Newtons) were used to calculate SWD values for each limb averaged across trials and expressed as a percentage of total BW. Thoracic and pelvic limb SWD were generated by combining values for left and right limb pairs. Left and right SWD were also expressed as a percentage of total thoracic or pelvic limb BW, respectively.

### Statistics

Analysis was performed using Jmp Pro 13^5^. Summary statistics (mean and SD) were calculated for thoracic to pelvic limb SWD (B2, B4, K2, K4, PSW) and left to right SWD between the thoracic and pelvic limbs (B4, K4, PSW). Comparison of the mean thoracic and pelvic limb SWD between the PSW and each of the scale combinations was determined using an ANOVA. Asymmetry between left to right for the thoracic and pelvic limbs, respectively, was compared between the B4, K4 and PSW methods using an ANOVA. Coefficients of variation were calculated to establish variability between trials for each scale combination. *P* < 0.05 was considered significant for all comparisons.

## Data Availability

The datasets used during the current study are available from the corresponding author on reasonable request.

## Abbreviations

B2: Two digital bathroom scales
B4: Four digital bathroom scales
BW: Body weight
IVDH: Intervertebral disc herniation
K2: Two digital kitchen scales
K4: Four digital kitchen scales
PSW: Pressure sensing walkway
SCI: Spinal cord injury
SWD: Static weight distribution

## Footnotes

1 Smart Weight Smart Tare Digital Body Weight Bathroom Scale, amazon.com

2 ETEKCITY Digital Kitchen Scale, Model No EK6212-S, amazon.com

3 Wingogh Anti Slip Tape, Safety Grit Tape 4” by 30FT, amazon.com

4 Tekscan High Resolution Animal Walkway System, Tekscan, Inc South Boston, MA, USA

5 SAS Institute, Cary, NC, USA
